# Comparing repeatability metrics for quantitative susceptibility mapping in the head and neck

**DOI:** 10.1007/s10334-025-01229-3

**Published:** 2025-03-01

**Authors:** Matthew T. Cherukara, Karin Shmueli

**Affiliations:** Department of Medical Physics and Biomedical Engineering, University College London, London, UK

**Keywords:** Magnetic resonance imaging, Reproducibility of results, Cancer of the head and neck

## Abstract

**Objective:**

Quantitative susceptibility mapping (QSM) is a technique that has been demonstrated to be highly repeatable in the brain. As QSM is applied to other parts of the body, it is necessary to investigate metrics for quantifying repeatability, to enable optimization of repeatable QSM reconstruction pipelines beyond the brain.

**Materials and methods:**

MRI data were acquired in the head and neck (HN) region in ten healthy volunteers, who underwent six acquisitions across two sessions. QSMs were reconstructed using six representative state-of-the-art techniques. Repeatability of the susceptibility values was compared using voxel-wise metrics (normalized root mean squared error and XSIM) and ROI-based metrics (within-subject and between-subject standard deviation, coefficient of variation (CV), intraclass correlation coefficient (ICC)).

**Results:**

Both within-subject and between-subject variations were smaller than the variation between QSM dipole inversion methods, in most ROIs. autoNDI produced the most repeatable susceptibility values, with ICC > 0.75 in three of six HN ROIs with an average ICC of 0.66 across all ROIs. Joint consideration of standard deviation and ICC offered the best metric of repeatability for comparisons between QSM methods, given typical distributions of positive and negative QSM values.

**Discussion:**

Repeatability of QSM in the HN region is highly dependent on the dipole inversion method chosen, but the most repeatable methods (autoNDI, QSMnet, TFI) are only moderately repeatable in most HN ROIs.

**Supplementary Information:**

The online version contains supplementary material available at 10.1007/s10334-025-01229-3.

## Introduction

Quantitative susceptibility mapping (QSM) is an advancing magnetic resonance imaging (MRI) technique that uses the phase of the MRI signal to reconstruct distributions of magnetic susceptibility [1–3]. QSM’s sensitivity to tissue composition through sources of magnetic susceptibility make it a potentially excellent tool for probing the pathophysiological changes associated with a range of different diseases. QSM has been shown to be sensitive to varying concentrations of iron and other metals deposited in tissue [4–6], as well as to changes in venous oxygenation [7] and tissue oxygen metabolism [8, 9]. Therefore, it is possible that QSM could provide a biomarker for tumour hypoxia, which is related to radiotherapy resistance and poor prognosis in many cancers [10], including head and neck squamous cell carcinoma (HNSCC) [11, 12].

Before investigating clinical applications of QSM, it is necessary to ensure that the quantitative results produced (namely, estimates of regional magnetic susceptibility $\chi$) are repeatable across multiple scans within the same subject, and lie within a normal range of biological variability for healthy, demographically similar subjects. In the brain, the repeatability of QSM results within subjects, on a test–retest model, has been demonstrated extensively [13–18]. Other studies have shown that reconstructed susceptibility values from the same subjects are consistent across sites [19], systems [20–22], and field strengths [23–25].

Methods for quantifying repeatability vary significantly throughout the literature. Many within-subject repeatability studies used a same-day or different-day test–retest model, acquiring two QSMs per subject, and comparing the results using Bland–Altman plots [15, 18, 26–29], linear regression [15, 17, 26, 29] and statistical tests such as the Wilcoxon signed-rank test [18, 28]. Several repeatability studies also calculated the intra-class correlation coefficient (ICC) between repeats [15, 17, 18], coefficient of variation (CV) [15, 16], and/or repeatability coefficient (RC) [14, 29]. When comparing different acquisition or reconstruction paradigms across multiple repeats, Lauzon and colleagues used a linear mixed effects model, with parameters for subjects and acquisition protocol variations [30], as did Naji et al. [22]. The variety of methods chosen to quantify the repeatability of QSM results suggests that there is not yet a clear consensus about what is expected or how different methods or protocols can be assessed. A statistical analysis of the different available metrics and their relevance to QSM may be warranted. Furthermore, many studies are limited in the range of statistical options available to analyse paired test–retest results. Testing the same subjects with multiple repetitions, across multiple dimensions of variation (different days, protocols, systems, locations), allows for a more comprehensive analysis of the sources of variation [19, 24, 30, 31].

The field of QSM body imaging is growing quickly and reproducibility studies have recently been performed for QSM in the vertebrae [27] and the liver [29]. Of major significance to the present study is the work of Karsa et al. in developing a repeatable pipeline for QSM in the head and neck [31]. The current study builds on that work, using the same multi-echo GRE data but now incorporating several of the most up-to-date QSM reconstruction algorithms available, and comparing their repeatability quantitatively using a variety of metrics, with the aim of establishing the one most suitable for this region and application.

While consensus exists within the QSM community on the kinds of algorithms that ought to be used for brain QSM, it is currently recommended that QSM acquisition and reconstruction protocols be optimized for other anatomical regions or applications on a per-study basis [32]. The conceptual (and practical) stages of most QSM reconstruction algorithms consist of: echo combination, phase unwrapping, masking, background field removal, and dipole inversion. As well as calculating quantitative metrics of reproducibility, QSM algorithms should also be evaluated on their ability to reconstruct visually artefact-free susceptibility maps, with contrast corresponding to anatomical expectations.

The head and neck (HN) region poses unique challenges for QSM, due to motion and flow artefacts, the presence of many air-tissue interfaces, and water-fat frequency shift. Previous work has optimized the sequence parameters used in acquiring the necessary 3D gradient echo (GRE) data, including in-phase echo times (TEs) that minimize fat–water artefacts, coronal acquisition, and a field of view (FOV) chosen to avoid wrap-around artefacts [33]. Previous work also investigated the effect of different algorithms on the initial stages of QSM reconstruction for HN data [34] (and see Supplementary Material in Karsa et al. [31]).

In the present study, we present an analysis of the intra-subject repeatability of six of the latest QSM dipole inversion algorithms in regions of interest (ROIs) in the brain and neck. Several metrics of repeatability are presented and their utility in comparing QSM values in these HN ROIs is discussed.

## Materials and methods

### Data acquisition

Ten healthy volunteers (seven female, age range 23–30 years) were recruited as part of a previous study [31], under local ethics committee approval. Subjects were scanned in two sessions one week apart, with three identical scans per session. Multi-echo 3D GRE images were acquired with a 3 T Achieva system (Philips Healthcare, Best, Netherlands) and a 16-channel HN receiver coil, using an optimized HN QSM sequence [31]. Sequence parameters are shown in Table 1. In-phase echo times were chosen to minimize chemical shift effects from fat [33].

**Table 1:** Sequence acquisition parameters

Parameter	Value
System	3 T Philips Achieva
Orientation	Coronal
Read-out direction	Head-Foot
Field-of-view (mm)	$240\times 240\times 220$
Resolution (mm)	$1.25\times 1.25\times 1.25$
Acceleration	SENSE $\times 2$ (first PE direction)
Repetition time $TR$ (ms)	22.0
Echoes	4
Echo times $TE$ (ms)	$4.61, 9.22, 13.83, 18.44$
Flip angle ($^\circ$)	12
Scan duration (min:sec)	6:04

Regions of interest (ROIs) were segmented from the first-echo GRE magnitude image from the first scan of each subject. Four deep-brain grey matter regions (thalamus, caudate nucleus, putamen, and globus pallidus) were segmented automatically using FSL FIRST [35]. Six head and neck ROIs (lymph nodes, submandibular gland, parotid gland, jugular vein, trapezius muscle, and a section of subcutaneous fat) were drawn manually in ITK-SNAP [36, 37] and FSLeyes [38]. Smaller, lower-contrast ROIs (lymph nodes and glands) were checked by an expert radiologist. ROIs were rigidly registered to later scans using transformations from FSL FLIRT applied to the first-echo magnitude images [39, 40]. For voxel-wise comparisons, the QSMs of subsequent scans were rigidly registered to the first acquisition in each subject in the same way.

### QSM Reconstruction

Complex multi-echo GRE data were denoised using Marchenko-Pastur principal component analysis (MP-PCA) [41]. Denoised complex data underwent nonlinear fitting to estimate field maps [42], as well as to provide an estimate of noise in each voxel.

Different options were explored for the subsequent steps in QSM reconstruction. First, the volume of interest must be masked from background voxels. For QSM in the brain, the QSM Consensus Organization Committee recommends segmentation of the volume of interest using an automated segmentation tool [32]; however, there is no automated method for segmentation of the whole HN region. Karsa and colleagues created a tissue mask from the reciprocal of the noise map, thresholded at 1.2 times the mean of the reciprocal of the noise [31]. In the present study, the holes in that mask were filled, and the mask was eroded by one voxel, corresponding to consensus recommendations [32]. This was compared against a noise-based mask without holes filled.

Since the measured phase can take only values in the interval $\left[\text{0,2}\pi \right)$, phase images contain abrupt “wraps” where adjacent voxels have values close to 0 and $2\pi$. Karsa et al. used Laplacian phase unwrapping (LPU) [43] to estimate the underyling phase [31]; however, this method is inexact, and the current consensus favours an exact phase unwrapping method. As such, we tested SEGUE [44], a region-growing unwrapping algorithm that preserves exact phase values [32].

Background field perturbations, resulting from sources outside the whole HN mask, must also be removed. Karsa et al. concluded that projection onto dipole fields (PDF) [45] was most repeatable. The brain QSM Consensus Committee recommends the use of V-SHARP [46], except in cases where $\chi$ values at the edge of the mask are very important, in which case PDF is preferred. Therefore, the repeatabilities of these two methods were compared.

A range of methods for dipole inversion have been proposed, many of which iteratively infer a susceptibility distribution, with the addition of a regularization term based on sparsity. Recently, several deep-learning-based dipole inversion algorithms have been proposed [47, 48]. It is also possible to perform dipole inversion simultaneously with background field removal by estimating local and background susceptibility sources iteratively. This technique is referred to as total field inversion (TFI) [49] and has been applied in other body regions where fat off-resonance effects are a major factor [50].

Six open-source dipole inversion algorithms were tested. These included iterative minimization with Tikhonov-regularization (iterTik) [31], which was the optimal method found by Karsa and colleagues [31], and five other state-of-the-art methods which were not previously tested. Fast non-linear dipole inversion (FANSI) [51] regularized by total variation was chosen, as it was one of the best-performing methods in the 2019 ‘QSM reconstruction challenge 2.0’ [52]. Two other state-of-the-art spatial-domain iterative reconstructions, which conform to consensus recommendations for brain QSM [32], were also tested: StarQSM [53] and automatically regularized non-linear dipole inversion (autoNDI) [54]. One available pre-trained deep neural network inversion method was tested (QSMnet) [47], as was a single-step iterative TFI method, preconditioned using ${R}_{2}^{*}$ values [49] (which were estimated from multi-echo GRE magnitude data using ARLO [55]).

### Repeatability analysis

Several metrics of repeatability have been applied to QSM data in the literature, which have differing degrees of utility given that susceptibility ($\chi$) distributions in biological tissue have both positive and negative values and means close to zero. When evaluating a quantitative biomarker, the Quantitative Imaging Biomarker Alliance (QIBA) recommends considering and reporting several metrics [56]. Therefore, we chose and reported several repeatability metrics for this QSM study, which are listed in Table 2 and described below [57]. Intra-session and inter-session repeatability within subjects was not considered in the present study. Instead, we focused on analyses of within-subject and between-subject variation. All statistical analyses were performed using MATLAB, version R2023a (MathWorks, Natick, MA).

**Table 2:** Metrics of Repeatability Used

Metric	Domain
Normalized root mean square error (NRMSE)	Voxelwise
XSIM	Voxelwise
Within-region standard deviation ${\sigma }_{r}$	ROI-based
Within-subject standard deviation ${\sigma }_{w}$	ROI-based
Between-subject standard deviation ${\sigma }_{b}$	ROI-based
Within-subject coefficient of variation CV	ROI-based
Intraclass correlation coefficient ICC	ROI-based

Voxel-wise measures of similarity across repeats within each subject were calculated for deep brain regions of interest. These were normalized root mean squared error (NRMSE), which was calculated by dividing the ${{\ell}}_{2}$ norm of the difference between two measurements by the ${{\ell}}_{2}$ norm of the first measurement, and XSIM, an implementation of the structural similarity index measure (SSIM) optimized for comparison of QSMs [58]. For NRMSE, lower values indicate better repeatability; whereas for XSIM, higher values indicate greater structural similarity, hence better repeatability.

After calculating NRMSE and XSIM in each ROI for each scan, repeated measures analysis of variation (RANOVA) was used to ascertain whether there were significant differences in these metrics between QSM methods, across all subjects and repetitions. An $F$-statistic was calculated for each test condition, and these were used to generate $p$-values to assess statistical significance. RANOVA makes the assumption of equal variance across different dimensions of variation (sphericity). Mauchly’s sphericity test was used to test this assumption, and in cases where sphericity was violated, the lower bound on the $p$-value was used instead of the original $p$-value estimate [59].

For every subject, scan, and method, mean $\chi$ (in ppm) in each ROI was calculated, along with the standard deviations across three dimensions of variation:

Within-region standard deviation ${\sigma }_{r}$ was defined as

1 $${\sigma }_{r}=\sqrt{\frac{1}{{N}_{v}}\sum_{v=1}^{{N}_{v}}{\left({\chi }_{v}-\overline{\chi }\right)}^{2}}$$

where ${N}_{v}$ is the number of voxels in the given ROI, ${\chi }_{v}$ is the susceptibility of voxel $v$, and $\overline{\chi }$ is the mean susceptibility in the ROI.

Within-subject standard deviation ${\sigma }_{w}$ was defined as

2 $${\sigma }_{w}=\sqrt{\frac{1}{{N}_{r}}\sum_{r=1}^{{N}_{r}}{\left({\chi }_{r}-\overline{{\chi }_{r}}\right)}^{2}}$$

where ${N}_{r}=6$ is the number of repetitions per subject, ${\chi }_{r}$ is the mean susceptibility calculated across all voxels (equivalent to $\overline{\chi }$ in Eq. 1) in the given ROI, for repetition $r$, and $\overline{{\chi }_{r}}$ is the mean ROI susceptibility across repetitions for the given subject.

Between-subject standard deviation ${\sigma }_{b}$ was defined as

3 $${\sigma }_{b}=\sqrt{\frac{1}{{N}_{s}}\sum_{s=1}^{{N}_{s}}{\left({\chi }_{s}-\overline{{\chi }_{s}}\right)}^{2}}$$

where ${N}_{s}=10$ is the number of subjects, ${\chi }_{s}=\overline{{\chi }_{r}}$ is the mean ROI susceptibility across all repetitions for the given subject $s$, and $\overline{{\chi }_{s}}$ is the mean of $\overline{{\chi }_{r}}$ across subjects.

It is expected that ${\sigma }_{b}$ (representing biological variations between individuals) will be greater than ${\sigma }_{w}$ (representing fluctuations across time within the same individual). Standard deviation is a useful metric of the repeatability of a measurement because it is defined in the same units as the measurement and can be corrected to avoid bias based on the number in a sample. However, the standard deviation depends on the mean value of the measurement and is therefore not directly comparable across methods which might produce different mean values.

RANOVA was used to test whether ${\sigma }_{w}$ and ${\sigma }_{b}$ were significantly different in each ROI, and whether the dipole inversion method used produced significantly different distributions of results.

Some studies report the repeatability coefficient (RC) of a measurement [14, 29]. This is defined as $RC=1.96\sqrt{2{\sigma }_{w}^{2}}$, and therefore scales the within-subject standard deviation to quantify the smallest significant difference that could be observed between two repeated measurements, at a 95% confidence interval [57]. We focused our analysis on (unscaled) ${\sigma }_{w}$ rather than RC, as the former is more directly comparable with other standard deviations. The within-subject coefficient of variation (CV) is a commonly reported metric in MRI repeatability studies, as its dimensionless nature enables direct comparison between measurements with different means [15, 16, 60]. It is given by

4 $$CV=\frac{{\sigma }_{w}}{\overline{{\chi }_{r}}}$$

which involves division by the mean, meaning that CV is vulnerable to taking extreme values if the mean is close to zero.

A final and commonly used quantifier of repeatability is the intra-class correlation coefficient (ICC), which is the ratio of between-subject variance to total variance:

5 $$ICC=\frac{{\sigma }_{b}^{2}}{{\sigma }_{b}^{2}+{\sigma }_{w}^{2}}$$

with sample variances calculated using ANOVA [61]. The one-way ICC model was chosen because there is no expectation of a systematic bias across repetitions, given that they are repeated acquisitions under the same conditions with the same equipment and protocol. ICC is dimensionless and enables comparisons between measurements with different means and ranges. A high ICC suggests that the variance present within measurements is due to genuine inter-subject differences rather than random variation across repetitions. Thresholds for classifying methods as repeatable or highly repeatable based on ICC vary in the literature, although Koo and Li recommend considering values greater than 0.75 to be indicators of ‘good’ reliability, and ICC greater than 0.9 would indicate ‘excellent’ reliability [62].

Confidence intervals (at 95%) were generated for estimates of ICC, using a bootstrapping technique with 1000 repetitions.

## Results

Images from all subjects were visually inspected for artefacts throughout the reconstruction process, and all pipelines resulted in visibly normative QSMs in the brain. There was considerable variation of the visual quality and streaking artefact prevalence in the HN region, likely due to the air-tissue interfaces and the larger (paramagnetic and diamagnetic) $\chi$ values in this region compared with a healthy brain.

The initial QSM reconstruction pipeline stages (masking, phase unwrapping, and background field removal) were assessed and the previously optimized HN QSM pipeline [31] was compared with pipelines incorporating consensus recommendations for brain QSM at 3 T [32]. Methods were compared using voxel-wise similarity metrics (NRMSE and XSIM) as well as ROI-average-based metrics (${\sigma }_{w}$ and ICC). The full results of these comparisons can be found in Supplementary Information 1.

The filling of holes in the HN mask (per consensus recommendations) did not significantly affect the repeatability of the resulting QSMs. Nor did the choice of phase unwrapping method (comparing LPU and SEGUE). On the basis of NRMSE, PDF background field removal resulted in more repeatable results than V-SHARP, although ${\sigma }_{w}$ was larger for PDF. On the basis of these results, the following choices were made for all subsequent QSM reconstructions: noise-based masking with holes filled, exact phase unwrapping with SEGUE, and background field removal using PDF.

**Fig. 1:**
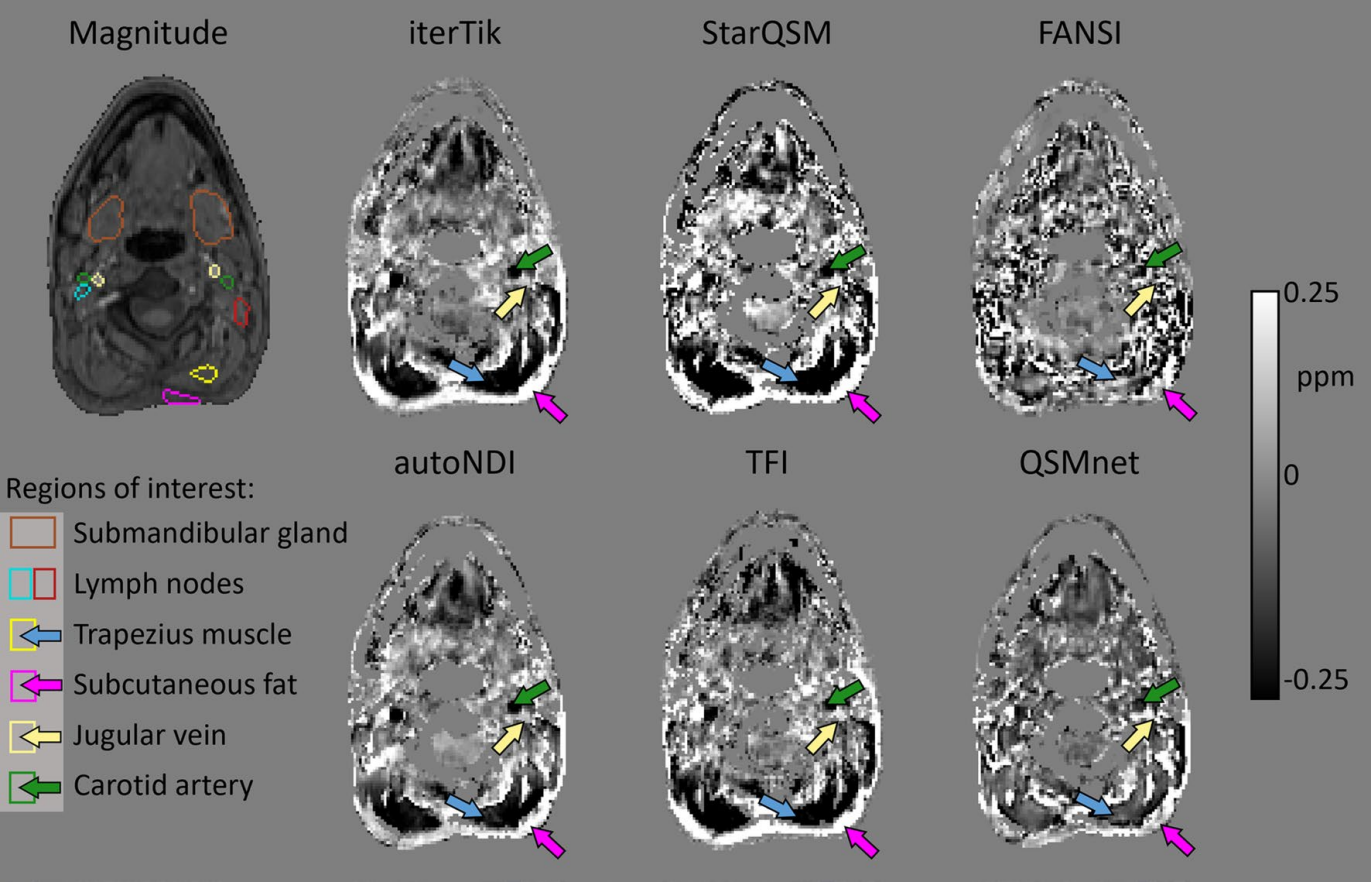
First-echo GRE magnitude image and QSMs from a representative subject, axial view at spinal vertebra C2, with several HN ROIs shown (parotid gland not shown, as it is in a different axial slice) .In most QSMs, trapezius muscle (blue arrow) appears diamagnetic, while subcutaneous fat at the back of the neck (pink arrow) appears paramagnetic. Similarly, jugular veins (yellow arrow) appeared paramagnetic and carotid arteries (green arrow) appear diamagnetic

**Fig. 2:**
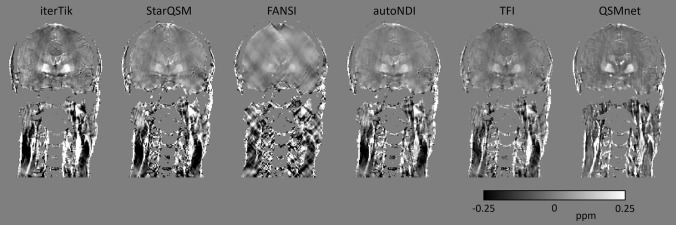
QSMs from a representative subject, coronal view. In the brain, iterTik and TFI maps are affected by residual background fields emanating from the top of the brain. FANSI suffers from significant streaking artefacts throughout. iterTik and QSMnet appear to show the strongest contrast in the deep-brain grey matter regions. There is a pronounced difference in contrast between QSMnet and the iterative methods in the neck

Figure 1 shows an example of axial QSMs from a single subject reconstructed with the six compared dipole inversion methods. Figure 2 shows coronal-view QSMs in a single subject, with all six dipole inversion methods. iterTik, StarQSM, autoNDI, and TFI share many visual similarities. The FANSI reconstruction is most affected by streaking artefacts throughout the neck. These artefacts may be removed or mitigated by increasing the weighting given to the total-variation regularization term; however, when this was tested, the resulting QSMs appeared over-regularized in the brain with almost no contrast. The results presented and used in subsequent comparisons used a regularization weight obtained by L-curve optimization calculated over the whole HN mask. The QSMnet reconstruction (the only one based on a deep learning method) shows less contrast between subcutaneous fat (orange arrow in Fig. 1) and muscle (blue arrow in Fig. 1)  than the iterative methods.

**Fig. 3:**
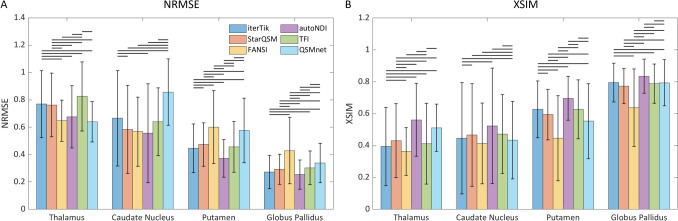
Mean voxel-wise similarity metrics across subjects, calculated in four brain ROIs. **A** NRMSE, where lower values indicate smaller errors between repetitions. **B** XSIM, where higher values indicate greater similarity between repetitions. Error bars indicate inter-subject standard deviations. Horizontal lines indicate pairs of methods that are significantly different ($p<0.05$)

Voxel-wise metrics of similarity (NRMSE and XSIM) were calculated across repeats within the four brain ROIs, for six dipole inversion methods. The results are shown in Fig. 3. Based on NRMSE, autoNDI performed best in three ROIs and showed no statistically significant difference in performance to FANSI and QSMnet in the fourth (thalamus). Based on XSIM, autoNDI outperformed the other metrics in every ROI, although other metrics performed equivalently well in certain ROIs (QSMnet in the thalamus; StarQSM and TFI in the caudate nucleus; iterTik in the globus pallidus).

**Fig. 4:**
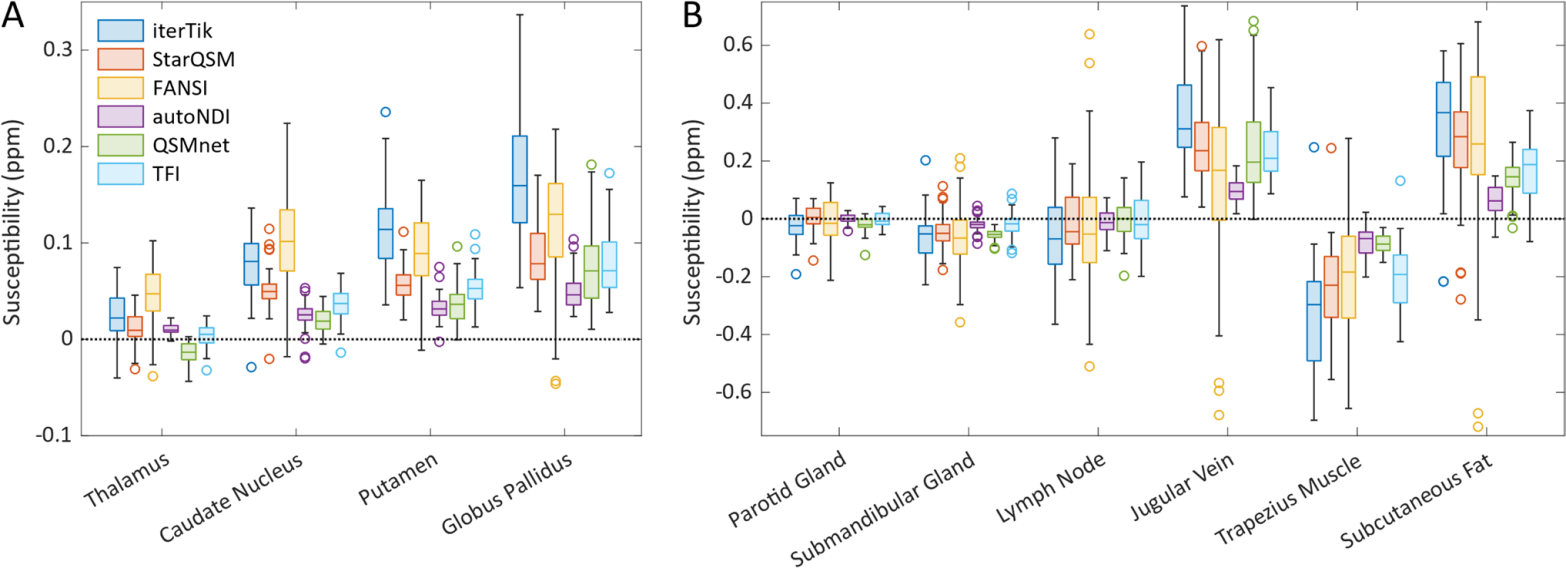
Box plots showing the distribution of mean $\chi$ values in each ROI across all subjects and acquisitions, for the six dipole inversion methods. **A** Brain ROIs. **B** Neck ROIs. Note that the y-axis susceptibility scales are not equal

Mean $\chi$ across all subjects and acquisitions, for each ROI and inversion method, are shown in Fig. 4. There is significant variation in the distribution of values in each ROI between the six tested dipole inversion methods, with StarQSM, autoNDI, QSMnet, and TFI generally resulting in the smallest range of values, and iterTik and FANSI the largest. iterTik and FANSI also tended to produce $\chi$ values of the greatest magnitude, while other methods, especially autoNDI and QSMnet, resulted in $\chi$ values closer to 0.

**Fig. 5:**
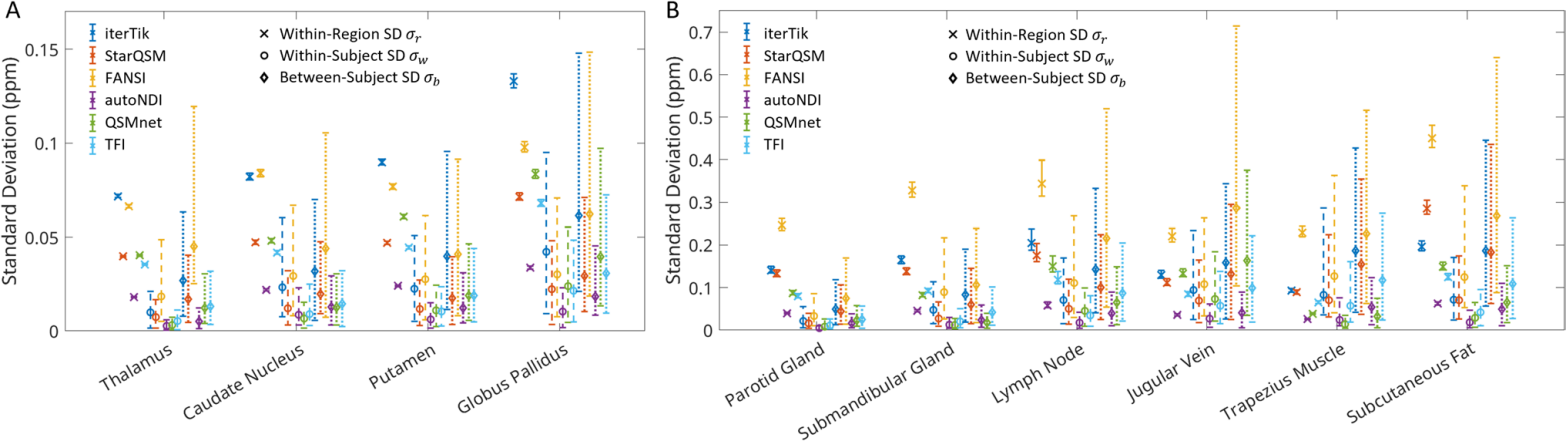
Interval plots showing voxel-wise standard deviation in each ROI (blue), standard deviation of ROI means across repetitions (orange), and standard deviation of ROI means across subjects (yell0w), with 95% confidence intervals. **A** Brain ROIs. **B** Neck ROIs. Note that the y-axis susceptibility scales are not equal

Figure 5 compares $\chi$ standard deviations in each ROI across three dimensions of variability: (1) within-region (voxel-wise) standard deviation ${\sigma }_{r}$; (2) standard deviation on the regional means, across six repetitions ${\sigma }_{w}$, within each subject; (3) standard deviation on regional means, across all subjects ${\sigma }_{b}$. 95% confidence intervals are shown. In most ROIs and for most methods, ${\sigma }_{r}$ was larger than both ${\sigma }_{w}$ and ${\sigma }_{b}$.

RANOVA within each dipole inversion method showed that ${\sigma }_{b}$ was significantly greater than ${\sigma }_{w}$ for all 6 methods across all 10 ROIs, with the single exception being FANSI reconstruction in the submandibular gland.

**Fig. 6:**
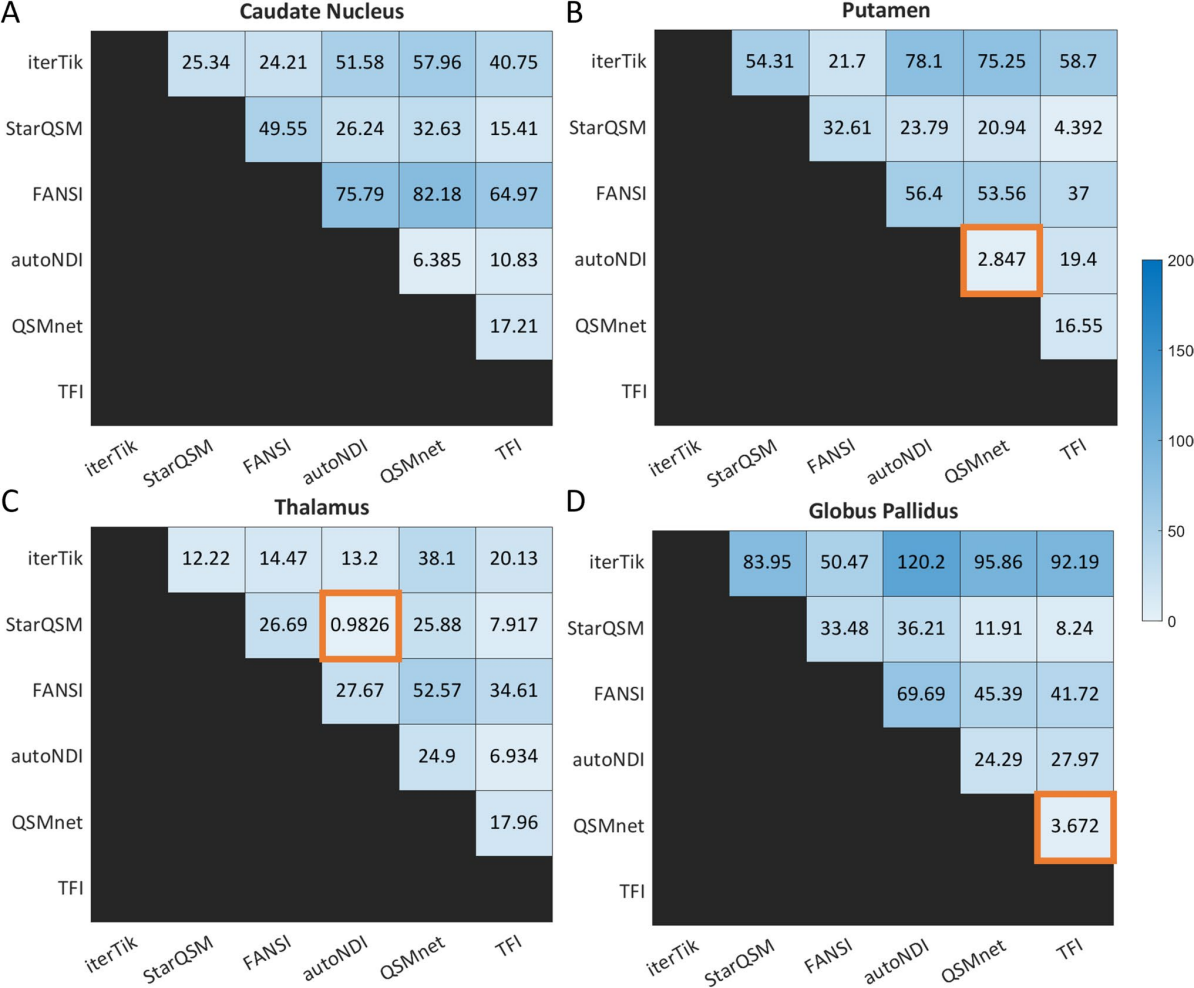
RANOVA results for the brain ROIs. The numbers are the mean difference (in ppb) of the ROI values between each method. The cells that are highlighted in orange are NOT statistically significant ($p>0.05$)

RANOVA was also used to assess whether variance within methods (across subjects and repetitions) was greater than the variance between methods. The results of this analysis in brain ROIs are shown in Fig. 6. In almost every case, the variance between dipole inversion methods was statistically significant, showing that the choice of method significantly affects the $\chi$ values obtained in the brain.

**Fig. 7:**
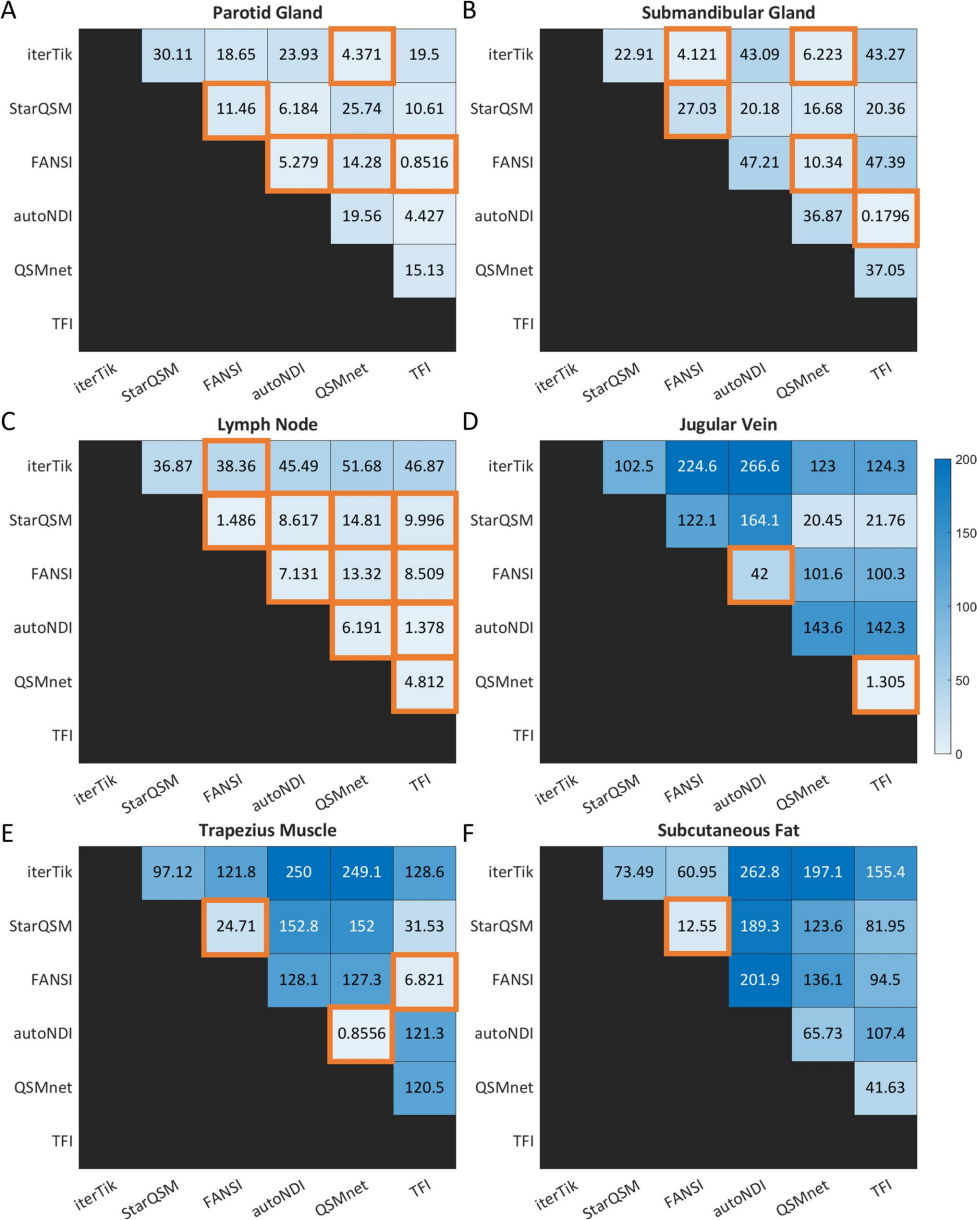
RANOVA results in the neck ROIs. The numbers are the mean difference (in ppb) of the ROI values between each method. The cells that are highlighted in orange are NOT statistically significant

The same analysis was performed for six neck ROIs, and the results are shown in Fig. 7. There is more variability between and within subjects in these ROIs compared to those in the brain, and so in some cases (such as the lymph nodes – Fig. 7c) the choice of dipole inversion method does not significantly affect the $\chi$-values obtained, although in the other neck ROIs (especially jugular vein, muscle, and fat – Fig. 7d–f) there are significant differences between almost all pairs of methods.

**Fig. 8:**
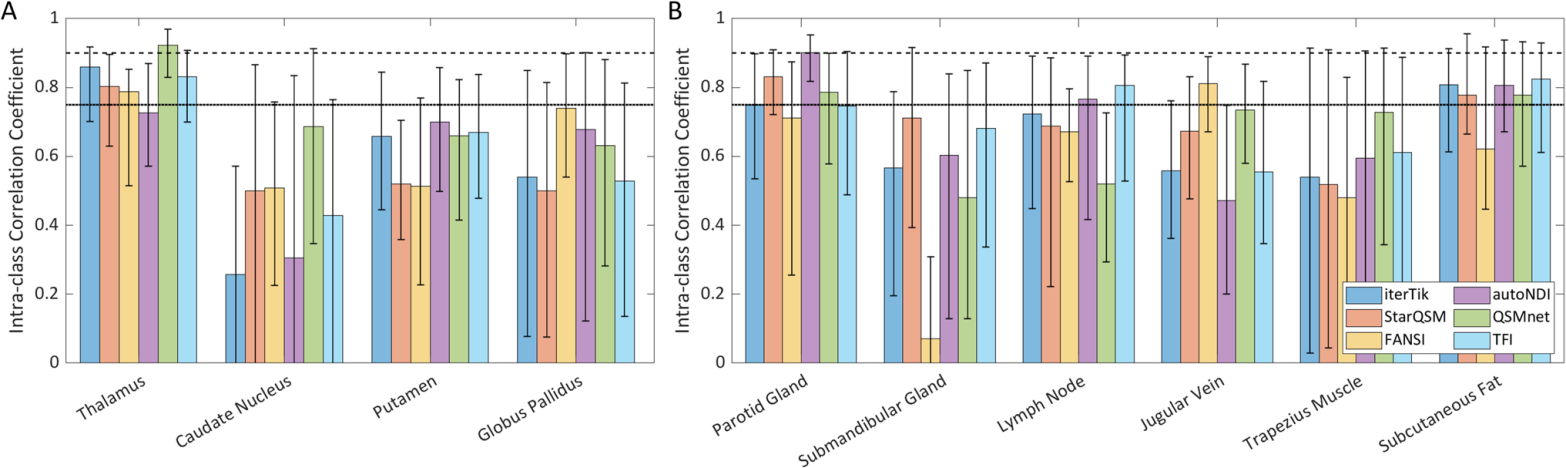
Intra-class correlation coefficient (ICC) in 10 ROIs for 6 QSM dipole inversion methods, where values greater than 0.75 indicate ‘good’ repeatability (dotted line), and values greater than 0.9 indicate ‘excellent’ repeatability (dashed line), with 95% confidence intervals shown as error bars. **A** Brain ROIs. **B** Neck ROIs

ICC results for the ten HN ROIs are shown in Fig. 8. These suggest good repeatability (ICC $>0.75$) from most methods in the thalamus only, while other brain ROIs are at best ‘moderately’ repeatable [62] (Fig. 8a). In neck ROIs (Fig. 8b) autoNDI performs best, with good repeatability in three of six ROIs. StarQSM, QSMnet, and TFI showed good repeatability in two neck ROIs each. The wide 95% confidence interval bars for ICC show that there is little variation between dipole inversion techniques in terms of their repeatability.

In many cases, CV calculations resulted in values with a magnitude greater than 1, probably a result of the mean $\chi$ being so close to zero in many ROIs. In light of this, CV was excluded from these analyses as a measure of repeatability.

## Discussion

### Metrics of repeatability

QSM is increasingly being applied to areas of the body outside the brain [29, 50, 63–65], for a better understanding of basic physiology as well as diseases such as cancer [66, 67]. As there are a multiplicity of methods for QSM reconstruction, and, as yet, no consensus on recommended techniques outside the brain [32], it is crucial to develop robust methods for assessing the repeatability of QSM methods in different anatomical regions.

The present study has built upon previous research into the repeatability of QSM in the head and neck [31]. In that work, the authors compared algorithms for different stages of the QSM reconstruction pipeline in terms of intra-session and inter-session repeatability across subjects, in ROIs in the brain and neck. This study has compared those findings with several up-to-date algorithms and implemented current consensus recommendations, and has compared repeatability within subjects and between subjects using a range of recommended and commonly used repeatability metrics.

It was found that some metrics were more informative and comparable than others. Coefficient of variation CV, in particular, was found in several neck ROIs to have a value greater than 1, whereas some literature sources measured CV of QSM in deep-brain ROIs as between 0.01 and 0.25 [18, 60]. In common with the findings presented here, Salluzzi and colleagues also encountered CVs greater than 1 in certain ROIs [16]. This accords with the expectation that CV is only valid as a measure of repeatability for strictly positive parameters whose mean is not close to zero [57, 68]. In light of this, CV was not used as a further comparison of repeatability in this study.

Intraclass correlation coefficient ICC was calculated for the different methods in each ROI. The 95% confidence intervals in ICC values (Fig. 8) are very broad in most cases, showing that there is little difference between methods in this regard. Using a threshold of ICC $>0.75$ as an indicator of ‘good’ repeatability [62], the best-performing method (autoNDI) produces repeatable results in only 3 of 10 ROIs. However, it should be noted that thresholds for ICC values vary in the literature. Ippoliti et al. consider ICC $>0.6$ to be ‘good’ and ICC $>0.75$ to be ‘excellent’ [24]. By this standard, QSMnet results are repeatable in 8 ROIs, and autoNDI and TFI in 7 ROIs each. The high repeatability of the QSMnet results is what might be expected from a deep learning method; however, it should be noted that the network was trained on brain data, which is expected to contain less $\chi$ contrast than the HN region [47].

When using ICC as a metric of repeatability, care should be taken as its value can be skewed high if between-subject variance is much greater than within-subject variance [61]. RANOVA analysis and comparison of standard deviations (Fig. 5) show that, while between-subject variance is greater than within-subject variance, the difference is small and not always statistically significant.

Costa-Santos and colleagues recommend that, in light of the limitations of both methods, limits of agreement (based on RC, which is linearly related to ${\sigma }_{w}$) and ICC should be used in combination when assessing repeatability or agreement [69].

Voxel-wise measures of image similarity are typically used when comparing methods against a known ground truth [52, 70]. Milovic and colleagues found that some of these measures (such as SSIM) do not appropriately account for the presence of both positive and negative $\chi$ values, and are therefore less valid when applied to QSM data [58]. The XSIM metric [58] is a better metric of similarity between QSM data and ground truth, and can therefore also be applied to assessing the similarity between pairs of QSMs. When comparing repeatability metrics, there was a correlation between voxel-wise measures (XSIM and NRMSE) and ROI-based methods (${\sigma }_{w}$ and ICC) (Figs. 3, 5 and 8).

### Repeatability of QSM reconstruction methods

Methodological choices for the initial stages of the QSM reconstruction pipeline were compared, based on previous findings and brain QSM consensus recommendations [31, 32]. Based on NMRSE, XSIM, ${\sigma }_{w}$, and ICC, the inclusion of holes in the noise mask made minimal difference to repeatability. Similarly, the choice of phase unwrapping method, between Laplacian unwrapping and an exact method (SEGUE) did not significantly affect repeatability in most ROIs. There were some differences in repeatability resulting from changes in the background field removal method, and generally PDF (as suggested by the previous study) [31] performed better than V-SHARP (consensus recommendation for brain QSM). These results are shown in Supplementary Information 1.

When comparing dipole inversion methods, the results presented above suggest that autoNDI and QSMnet produced the most repeatable results throughout the HN region. autoNDI is a non-linear iterative, regularized method, and therefore shares similarities with FANSI, and to a lesser degree with other iterative methods like iterTik and StarQSM. Its advantage is in the use of magnitude data as a prior, abrogating the need for fine-tuning of regularization parameters [54]. QSMnet is a deep-learning-based dipole inversion method, which, while producing repeatable results, has important limitations that must be borne in mind. Crucially, the model was trained on brain data only, acquired at a different resolution from that used in this study (1 mm isotropic, as opposed to 1.25 mm isotropic) [47]. The resulting susceptibility maps (Figs. 1 and 2) and ROI $\chi$ values (Fig. 4) tend to exhibit less contrast than other methods, perhaps reflecting the generally lower contrast in the brain, compared to what would be expected in the neck or other parts of the body. When dealing with naturally heterogeneous tissues, or tissue in the presence of disease, care must always be taken when interpreting the results of models that were trained on healthy, more homogenous, data.

### Sources of variation in QSM results

As well as producing results that are repeatable across multiple acquisitions in the same subject, QSM reconstruction methods must be reproducible across acquisition parameters (including systems, vendors, and field strengths [19, 24, 25]) and directly comparable between demographically similar subjects. This is necessary for cohort-level analyses between patients and healthy controls that are common in clinical applications studies [71–73].

In QSM, as in other quantitative imaging modalities, variation in quantitative values can occur over several scales. First, even within relatively small anatomical regions of interest (such as deep grey matter nuclei, or lymph nodes in the neck) there is significant heterogeneity of tissue. This inherent, biological, source of variation can be probed with texture-based analyses and has been shown in some cases to have clinical relevance [74, 75], but is most often ignored by taking only mean or median values from a ROI. In QSM, texture-related variation may also be the result of streaking artefacts caused by failures in dipole inversion. These can be mitigated by appropriate masking and regularization of dipole inversions and should be caught by visual quality control [32].

Even in results that are free from visually discernible artefacts, such as maps from autoNDI QSM (see Fig. 2), the within-region standard deviation was significantly greater than the variation of the means between acquisitions, in all but three ROIs (caudate, jugular vein, trapezius muscle—Fig. 3). This poses a potentially fundamental problem for identifying regions of pathology within such ROIs using QSM.

Another source of variation in QSM results is physiological variations that occur within subjects over time. It has been demonstrated that short-term and long-term changes in oxygenation impact susceptibility in the brain [76–78]. These variations ought to be captured by repeated measurements on the same subject over time and can be seen in the fact that intra-session repeatability is generally better than inter-session repeatability [16, 31]. Understanding and accounting for the scale of these variations is important for assessing the effect of disease and/or treatment on $\chi$ values.

A third level of variation occurs between individuals. RANOVA analysis showed that between-subject variation was greater than within-subject variation in every ROI for almost every method tested (Figs. 6 and 7). This suggests that there are genuine differences between individuals, even those who are healthy and drawn from a relatively narrow age range (23–30 years). Many QSM studies attempt to mitigate age- and sex-based differences by including these as factors in multivariate linear modelling [26, 79–81].

In order for QSM to be applicable in detecting or staging disease, the resulting change in $\chi$ must be greater than the variation that would be expected naturally, both within subjects and between demographically similar subjects. ${\sigma }_{w}$ can be used to calculate minimum detectable effect size and has been found to vary significantly between ROIs (although it is skewed by mean $\chi$ values in each ROI). In deep-brain grey matter ROIs (such as the putamen) a change in $\chi$ of 17 ppb would be detectable at 95% confidence, but this minimum increases to 49 ppb in the lymph nodes and 83 ppb in the jugular veins (for the most repeatable dipole inversion method, autoNDI).

RANOVA analysis showed that, in most ROIs, the greatest source of variation in $\chi$ was the choice of the dipole inversion method, corroborating previous results [82]. In the HN region, this choice significantly affects the possibility of detecting changes. Across all ten ROIs, the minimum detectable effect size was more than five times greater for FANSI than autoNDI, meaning that any disease-related $\chi$ effect would have to be five times larger to be detected. This highlights the importance of QSM reconstruction pipeline optimization, over and above acquisition protocol optimization, for every application [31–33, 63], although such efforts typically involve pipeline development in healthy individuals, and the assumption is made that the same image quality and repeatability obtained in healthy subjects will transfer to patient populations.

### Limitations

There are limitations to the analyses presented in this study. First, the data were acquired using the same scanner with the same parameters, for all six repetitions. This means it is not possible to assess inter-scanner or inter-sequence reproducibility of QSM reconstruction algorithms with these data. In addition, the HN region suffers from some additional artefacts which do not normally affect brain QSM. Chief among these is the effect of fat on the GRE signal, given its chemical shift relative to water. In this study, in-phase echo times were used to minimise phase differences between fat and water [33]; however, this practice assumes that the fat spectrum has a single peak. Adopting multi-peak in-phase echo times may offer a small reduction in bias, but this should not significantly affect repeatability [65]. Alternatively, a water-fat reconstruction method may improve the quality of QSMs in the HN region [83].

Our results showed that some ROIs (deep-brain grey matter regions, and larger glands in the neck) have much more consistency across acquisitions and between individuals, than other regions (such as lymph nodes or muscle tissue).

It is likely that choices of algorithm throughout the QSM pipeline will affect the accuracy of final $\chi$ distributions, as well as their repeatability. Given the absence of ground truth $\chi$ values in HN ROIs, it is beyond the scope of this work to determine whether some methods produce more accurate $\chi$ results than others.

## Conclusions and future work

In conclusion, for an imaging modality such as QSM to be of clinical use in a given application, such as head and neck cancer, it must be demonstrated that the quantitative results obtained are repeatable across acquisitions and comparable between individuals. Our study has quantified the repeatability of regional susceptibility measures in the HN region using several standard metrics and has used those to compare the results of several current dipole inversion algorithms.

It was found that joint consideration of within-subject standard deviation and intra-class correlation coefficient provided a good indicator of repeatability, as these two statistics have complementary strengths and limitations. Automatically regularized, non-linear iterative dipole inversion methods, such as autoNDI, produced the highest repeatability in most ROIs. A deep learning method (QSMnet) also showed promise but has limitations based on the training data that was used.

Although thresholds for acceptable repeatability are not easily transferred between modalities and applications, further work will be required to validate QSM as a clinically useful tool in the HN region. This work should include further investigation of deep learning QSM methods, including those specifically trained on HN data, and investigation of susceptibility source separation methods which may help to increase contrast and reduce heterogeneity within ROIs.

## Supplementary Information

Below is the link to the electronic supplementary material.

Supplementary file1 (DOCX 2150 KB)

## Data Availability

All MATLAB code used in generating the results reported in this study is available at https://github.com/Cherukara/HNRepeatability. Anonymized NIFTI data used for this study are available at M. Cherukara and K. Shmueli, “Head and Neck QSM Repeatability Data.” University College London. Dataset, 2024. 10.5522/04/27993215
