# Supplementary material for: Comparing repeatability metrics for quantitative susceptibility mapping in the head and neck
Source: MAGMA. 2025 Mar 1;38(3):449–63. doi: 10.1007/s10334-025-01229-3 (PMC12255659; doi:10.1007/s10334-025-01229-3)

**Supplementary Information 1**

**Supplementary Figure 1.** Effect of masking method on repeatability, as quantified by (A) NRMSE, (B) XSIM, (C) ICC. In two brain ROIs (caudate and putamen), the filled mask produces more repeatable results the noise mask, based on NRMSE and XSIM. In the HN ROIs, based on ICC, the results are mixed.


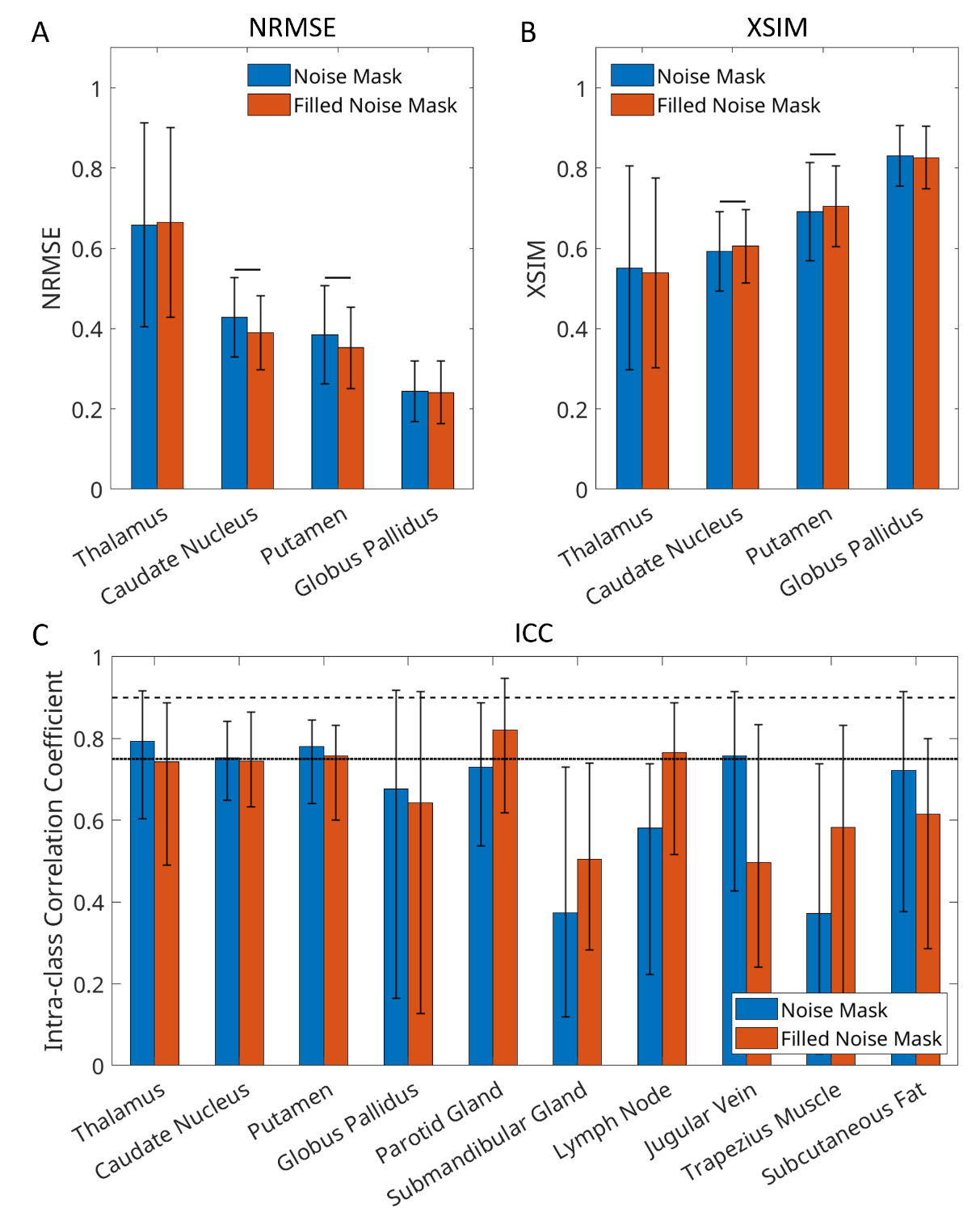


**Supplementary Figure 2.** Effect of phase unwrapping method on repeatability, comparing an approximate method (LPU) with a region-growing exact method (SEGUE). Quantified by (A) NRMSE, (B) XSIM, (C) ICC. Based on voxel-wise repeatability measures (NRMSE and XSIM) there are only minor differences between the methods in some ROIs. The regional repeatability metric (ICC) shows differences between the two methods in some ROIs, with LPU performing well in the caudate, putamen, and parotid gland). In addition to these, SEGUE demonstrated good repeatability in a lymph node ROI.


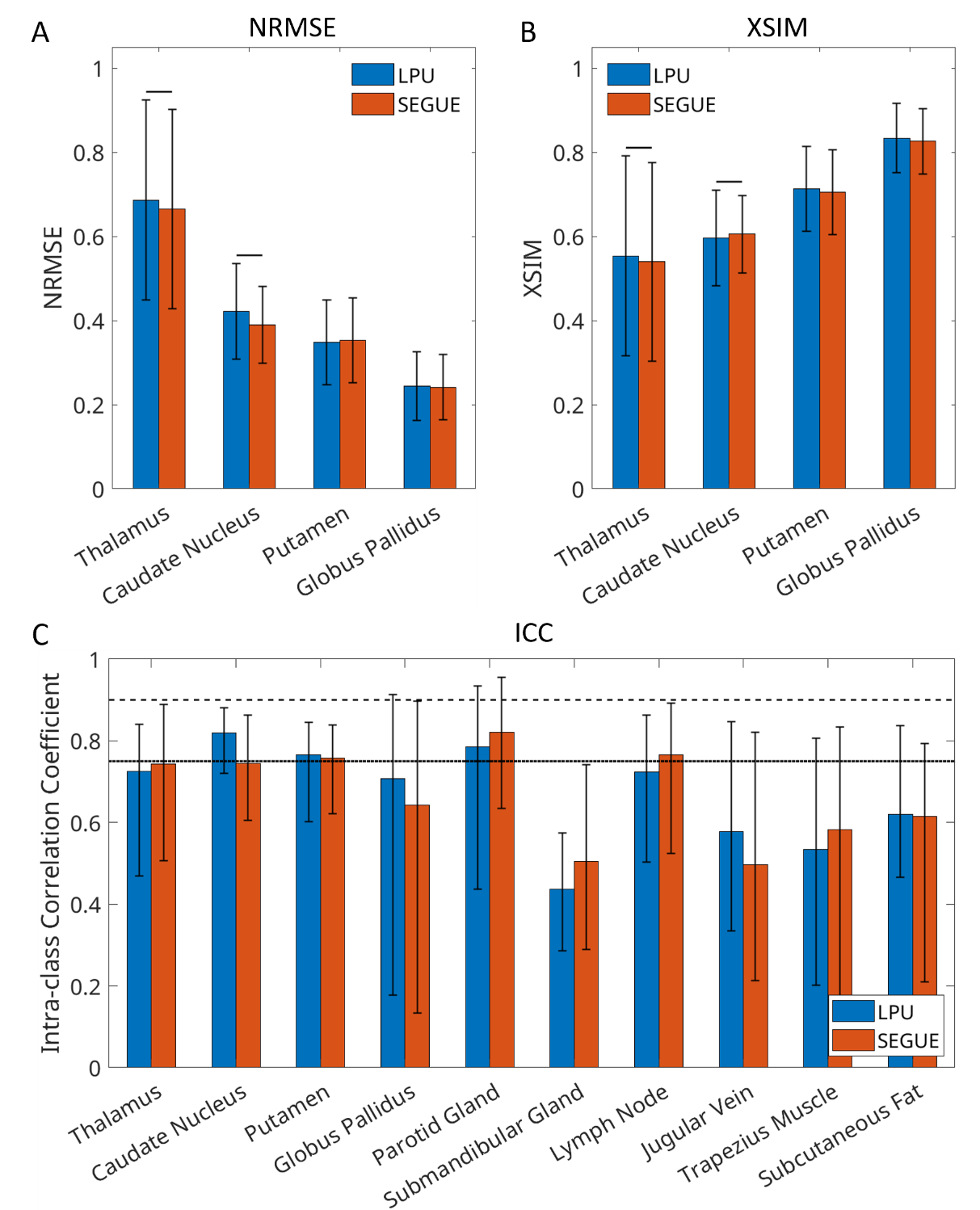


**Supplementary Figure 3.** Effect of background field removal method on repeatability, comparing PDF and VSHARP, quantified by (A) NRMSE, (B) XSIM, (C) ICC. Based on NRMSE, PDF consistently performs better than VSHARP across brain ROIs; however the XSIM results are mixed. PDF performs slightly better in ICC across ROIs, with good repeatability in 3 regions (putamen, parotid gland, lymph node), compared to VSHARP which returned good repeatability in only the parotid and lymph node. In most other ROIs, even though ICC was lower than 0.75, PDF was still more repeatable than VSHARP.


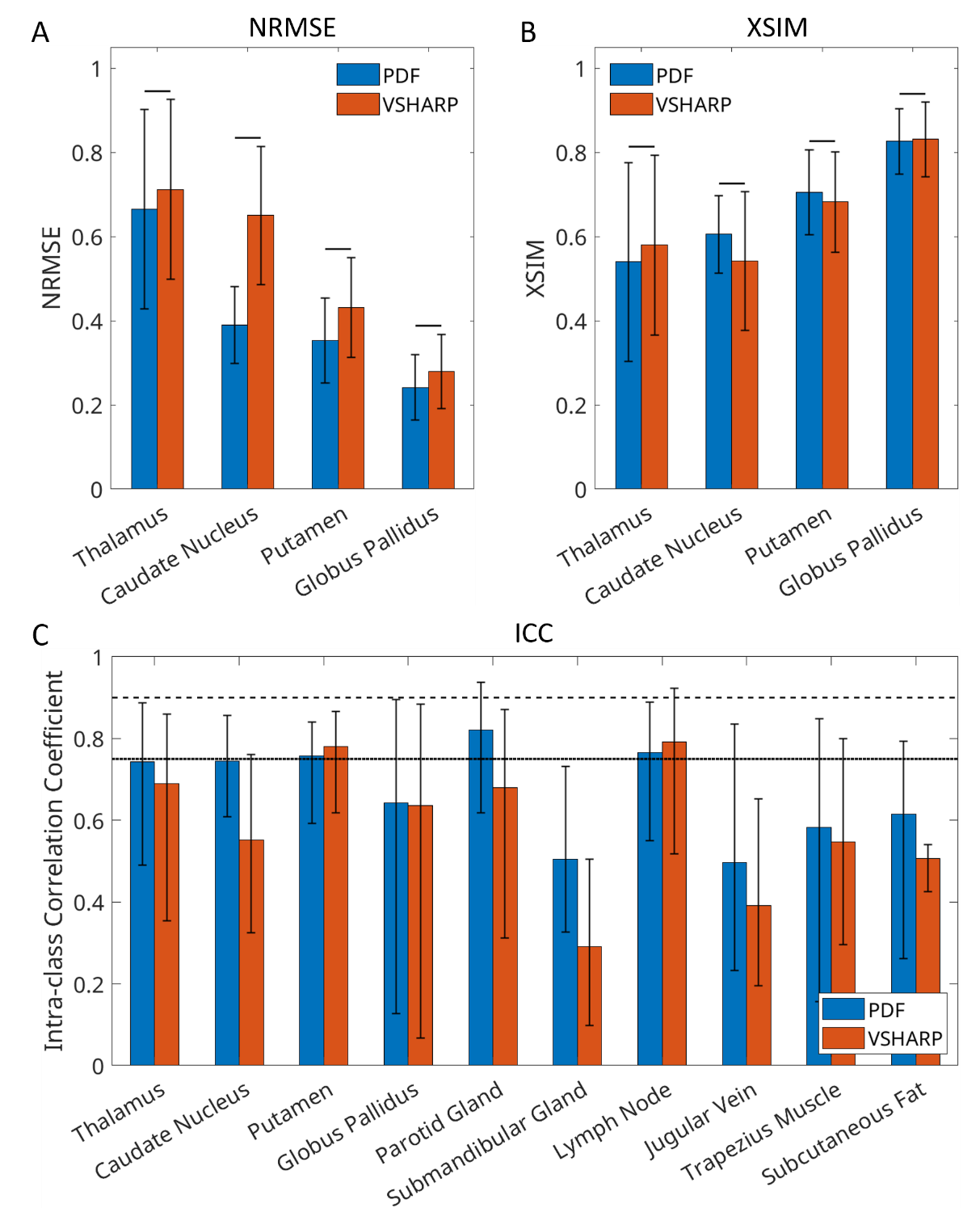

Supplement: Supplementary file 1 — Supplementary file1 (DOCX 2150 KB) [file 10334_2025_1229_MOESM1_ESM.docx]
